# Assessing mobile instant messenger networks with donated data

**DOI:** 10.1007/s13278-025-01550-8

**Published:** 2025-12-27

**Authors:** Rense Corten, Laura Boeschoten, Thijs Carrière, Stein Jongerius, Bella Struminskaya, Joris Mulder, Parisa Zahedi, Shiva Nadi Najafabadi, Adriënne Mendrik

**Affiliations:** 1 Department of Sociology/ICS, Utrecht University, Utrecht, The Netherlands; 2 Department of Methodology and Statistics, Utrecht University, Utrecht, The Netherlands; 3 Centerdata, Tilburg, The Netherlands; 4 Department of Research IT, Utrecht University, Utrecht, The Netherlands; 5 Eyra, The Hague, The Netherlands

**Keywords:** Online social networks, Data donation, Survey research, WhatsApp

## Abstract

Despite the increasing popularity and societal relevance of mobile instant messenger services (MIMSs) such as WhatsApp, Telegram and Signal, the vast majority of the research on social media is focused on the “traditional” social media platforms such as Twitter (X) and Facebook, mostly due to the difficulty of accessing MIMSs data. Consequently, little is known about even the most basic topological features of societal-scale instant messenger networks. To overcome this knowledge gap, we employ the innovative approach of data donation by respondents of a high-quality probability-based panel in the Netherlands (the LISS panel) to collect user data while preserving their privacy. Focusing on WhatsApp as the most popular MIMS, this study collects the first measurement of MIMS usage on a national probability sample, focusing on degree distributions as a key feature of network topology. We find that the degree distribution of contacts is best approximated by a log-normal distribution, while the distribution of group membership is best approximated by the exponential distribution. At the individual level, we find that predictors of degree derived from the literature on extended networks mostly replicate.

**Supplementary Information:**

The online version contains supplementary material available at 10.1007/s13278-025-01550-8.

## Introduction

Online social networks play an increasingly important role in contemporary societies, and as such have been extensively studied in the social sciences in recent years, for example for their role in civic and political participation (Boulianne 2018), the spreading of misinformation (Allcott and Gentzkow 2017), polarization (Bail et al. 2018), and social cohesion (González-Bailón and Lelkes 2023). By far, most of the research on online social networks focuses on the “traditional” social media platforms such as Twitter (now X) (Abdul Reda et al. 2021; Boulianne 2018; González-Bailón and Wang 2016), and, to a lesser extent, Facebook (Bond et al. 2012; Chan 2016; Harlow 2012; Valenzuela et al. 2018). Existing research thereby largely ignores *mobile instant messenger services* (MIMSs) such as WhatsApp, Signal and Telegram, which not only play an increasingly important role in, for example, social movements (Montag et al. 2015), but meanwhile rival social media platforms in popularity (Oberlo 2020). The relative popularity of MIMSs thus stands in sharp contrast to the available empirical knowledge on these platforms, giving these networks the status of “elephant in the room” in the field of social media research.

A key reason for the scarcity of research is that in contrast to traditional social media platforms, MIMSs are hardly accessible to researchers. Whereas social media platforms typically consist of networks of semi-public profile websites of users, which may be scraped or otherwise observed, MIMSs generally operate from users’ mobile devices, hidden from view. Moreover, even when data on interactions between users are collected by service providers (e.g., Meta, the owner of WhatsApp), data are typically proprietary and not available for research, leading to the paradoxical situation that although today more data on social interactions are being collected than ever before, one of the largest and potentially most consequential digital social networks at the moment is mostly a black box to (academic) researchers, while large corporations are in a much better position than academic researchers to capitalize to study these networks (Corten 2019; Lazer et al. 2020). In this paper, we aim to remedy this situation by means of a novel data set on WhatsApp networks in the Netherlands, collected by the innovative method of *data donation*. To our knowledge, our data provide the first empirical description of WhatsApp interactions for a probability sample of a national population, and also constitute one of the first applications of the data donation approach to such a sample.

Clearly, given the current lack of empirical knowledge, there are many open questions on even the most fundamental topological features of the WhatsApp network, such as the degree distribution, clustering, distances (e.g., small-world properties), community structure, etc. However, since our method is fundamentally based on a sample of *ego-networks* (or personal networks), we only have data on direct connections of the users in our sample, which prevents us from studying structural features that require information on longer distances, such as clustering or community structure. More than a limitation of our method, we argue that this is mostly due to the technical nature of WhatsApp, which makes the collection of data beyond direct connections exceptionally hard among large populations, for practical and ethical reasons that we will explain in more detail below.

We therefore focus on the statistical properties of the *degree distributions* of the user-to-user and the user-to-group networks to provide a first assessment of the topology of the WhatsApp network in the Netherlands. Degree distributions have been studied widely as a fundamental topological feature of large networks (Barabási 2016; Newman 2010), are the topic of foundational papers in the network science literature (Albert and Barabási 1999, 2002) and the subject of one of its most heated controversies (Broido and Clauset 2019; Holme 2019). Properties of degree distributions have been associated with underlying growth processes of networks (Albert and Barabási 1999; Jackson and Rogers 2007), robustness of network structures (Albert et al. 2000), and with dynamics of processes in networks, such as the spread of epidemics (Pastor-Satorras and Vespignani 2001) or the emergence cooperation (Santos and Pacheco 2005). While properties of degree distributions are thus a common starting point for comparison between large-scale networks (Broido and Clauset 2019; Clauset et al. 2009; Jackson and Rogers 2007), including online social networks (Ahn et al. 2007; Corten 2012), our paper is, to our knowledge, the first to estimate this distribution among a large population for the WhatsApp network.

Most of the vast literature on degree distributions focuses on one-mode networks; in comparison, the topological features of two-mode (or multi-mode) networks are relatively poorly understood even though such networks are commonplace (Guillaume and Latapy 2006). Due to the presence of the “groups” feature, the WhatsApp network can also be analyzed as a two-mode network (that is, the user-to-group network). We contribute to the still relatively scarce literature on two-mode networks by mapping the empirical degree distribution of the user-to-group network in WhatsApp.

Finally, at the individual level, variations in degree are associated with important outcomes such as mental and physical health, mortality, social support, and more generally, social capital (van Tubergen and Volker 2015; Völker et al. 2025). Studies on the sizes of personal networks have long been mostly restricted to networks of strong ties, due the difficulty of empirically measuring weaker ties (e.g., acquaintanceship networks). An emerging literature in sociology uses innovative statistical methods (e.g., the Network Scale-Up Method; McCormick et al. 2010) to approximate network sizes and to identify individual covariates of degree (Jeroense et al. 2025; Lubbers et al. 2019; Völker et al. 2025). Numbers of contacts in online networks such as WhatsApp, directly observed, provide a more *behavioral* measure of such extended networks, which does not rely on survey questions and complex statistical approximation via methods such as the Network Scale-Up Method. We thus contribute to the literature on extended networks by assessing the individual covariates of degree in both the user-to-user (one-mode) network and the user-to-group (two-mode) network. In the remainder of the paper, we first briefly introduce WhatsApp in the Netherlands as a research context. We then discuss related research and formulate our expectations based on this work. Next, we introduce our data collection and analytical approach, present our results, and conclude with broader implications and suggestions for further research.

### Research context: WhatsApp in the Netherlands

WhatsApp is a free instant messaging application for smartphones initially made available on the iPhone in 2009, and available on other operating systems, most notably Android, from 2010. It allows users to send text messages as well as images, video, files, and audio messages to other users of the app via the internet. While other web and desktop interfaces are also available besides the smartphone app, in principle a WhatsApp account is connected to a phone number (WhatsApp 2024a). Users can message any other user who is in their phone’s contact list. In contrast to typical social media platforms (Boyd and Ellison 2007), WhatsApp does not feature publicly searchable profile pages of users, nor does it allow users to view and traverse connections of other users.

Besides user-to-user messaging, a key feature of WhatsApp is the possibility to create *groups*, which are initiated by a single user but may include up to 1024 members (WhatsApp 2024b). Messages to the group are instantly distributed to all group members and occur within a single “chat” thread on the user interface. Other group members may be invited or added by the group initiator, while some public groups can also be joined unilaterally via a public “join link”. Recently added features include *channels*, which function as groups in which only channel administrators can “broadcast” messages to other group members (“followers”) but not vice versa, and *communities*, which combine multiple groups under a single “umbrella”.

Although WhatsApp was founded in the United States and is currently owned by the US-based technology corporation Meta (formerly Facebook), its popularity is relatively modest in the US as compared to the rest of the world, in particular in Latin America, parts of Asia, and Europe (Johns et al. 2023). In 2023, WhatsApp was reported to have around 13 million users in the Netherlands, on a population of nearly 18 million (Statista 2023).

## Related work and predictions

The scarce empirical research on instant messenger services focuses primarily on publicly accessible WhatsApp groups in political contexts (Bursztyn and Birnbaum 2019; Garimella and Tyson 2018; Resende et al. 2019), or relies on survey methods with small convenience samples (Agrawal 2020; Garimella et al. 2024; Rosenfeld et al. 2018; Seufert et al. 2016). A notable exception is Seufert et al. (2022), who rely on a relatively large sample of chat conversations donated by volunteers to study usage patterns. Research on the competing instant messenger service *Telegram* tends to be somewhat more comprehensive in terms of number of research participants due to the more open nature of the platform (e.g., Telegram features an API), but is still mostly restricted to public groups and channels (Dargahi Nobari et al. 2017; Kermani 2020), or focuses on relative fringe groups such as right-wing extremists rather than the general population (Urman and Katz 2020). Consequently, the existing research provides little guidance with regard to the overall network topology of instant messenger networks: we lack knowledge about even the most basic topological features of the societal-scale instant messenger network such as the degree distribution (number of contacts per user) or the degree of clustering, which will be strongly dependent on the group structure.

The literature on social media networks more broadly is vast and spans a large variety of disciplines, including (but not limited to) the social sciences, economics, computer science and physics. Consequently, we limit our discussion here to the two key interests of our paper: the overall degree distribution of social media networks and individual-level predictors of degree.

A key finding in the network science literature is that degree distributions of large networks tend to be heavy-tailed in the sense that there is a large heterogeneity in degree with many of the nodes having few connections, while other nodes have many connections and serve as “hubs” in the overall structure. A particularly strong finding in the early network science literature, and arguably one of the findings that sparked the field as a whole, was that a remarkable number of large networks display a particular type of heavy-tailed degree distributions, namely power law distributions (Barabási 2016). Theoretically, such “scale-free” degree distributions are predicted by a generative process that involves both growth and preferential attachment in which the probability for nodes to attract new connections is correlated with their degree (Albert and Barabási 1999, 2002). More recently, the universality of this finding has become somewhat controversial (Broido and Clauset 2019; Voitalov et al. 2019). In addition, research on *social* networks suggested that even if the power law claim holds for many types of biological and technical networks, social networks, including but not limited to social media networks, may be an exception (Jackson and Rogers 2007).

Although Facebook is probably the best-known social media network and accordingly has attracted a lot of attention from researchers (Boulianne 2018; Rains and Brunner 2015), studies of the degree distribution of the network are relatively scarce, due to the proprietary nature of the data required to answer such questions. Studies relying on privileged access to Facebook’s data (Ugander et al. 2011) or on probability samples of the Facebook population (Gjoka et al. 2010) find that while the degree distribution of “friendship links” on Facebook is heavy-tailed, it does not conform to a power law distribution but instead suggests “multi-scaling behavior” with multiple “regimes” in the degree distribution, possibly due to several types of nodes displaying different rates of connectivity. These findings are consistent with research on MSN (Leskovec and Horvitz 2008), the Dutch platform Hyves (Corten 2012), and the Korean platform Cyworld (Ahn et al. 2007). In the case of Twitter, which is a directed network, Meyers et al. (2014) report a power law distribution for indegree but a log-normal distribution for outdegree.

While it may thus seem somewhat naive to expect a power law degree distribution for the WhatsApp network, we nevertheless take this as a “baseline” expectation, given the prominence of this distribution in the literature and its supposed universality (cf. Barabási 2016). Alternatively, we consider the exponential distribution, which is predicted by a process that involves growth but not preferential attachment (Albert and Barabási 1999), and the log-normal distribution, which has been previously found in extended networks (McCormick et al. 2010).

Besides the user-to-user network of WhatsApp contacts, the “groups” feature of WhatsApp also creates a *user-to-group* network, which, more formally, constitutes a *bipartite* or *two-mode* network. Empirical research on two-mode networks is relatively scarce, possibly because studies of bipartite networks are often limited to one-mode projections of two-mode networks (e.g., the user-to-user network as implied by the user-to-group network). While for questions such as related to the spread of information between individuals (via groups) the user-to-user projection may, intuitively, indeed seem more relevant, analytical work suggests that the topological features such as the degree distribution of such one-mode projections crucially but non-trivially depend on the degree distributions (e.g., distributions of the number of groups per user and of the number of users per group) of the underlying two-mode network (Vasques Filho and O’Neale 2018). Empirical work that studies degree distributions of (online) social networks is relatively scarce, but tends to show heavy-tailed distributions, which may or may not follow power laws (Mitrović and Tadić 2012; Shang et al. 2010; Zhang et al. 2008). Given the absence of a well-established consensus in the literature about plausible specific degree distributions, we again consider the power-law distribution, the exponential distribution, and the log-normal distribution.

Research on what determines personal network size in online social networks is scarce in general, and as to be expected, WhatsApp is no exception. However, there is a growing literature on the determinants of the sizes of personal networks more generally. In particular, this literature aims to assess the sizes of *extended* personal networks, that is, personal networks beyond strong ties (Fu 2005; Hill and Dunbar 2003; Hofstra et al. 2021; Lubbers et al. 2019). Given evidence that the structure of online social networks tends to mimic the structure of “offline” networks (Dunbar 2016; Dunbar et al. 2015; Hofstra et al. 2017; Van Zalk et al. 2014) and given the absence of theory on instant messenger networks more specifically, we rely on this literature to derive expectations on the determinants of network sizes in the WhatsApp network.

The available theory on extended personal network size, in turn, borrows most of its argumentation from the literature on *strong* ties, which typically takes a choice-constraints approach, that is, it assumes that connections in social networks result from choices made by individuals under (social) constraints (Fischer et al. 1977). On the “choice side”, relevant mechanisms include variations in cognitive ability to maintain social networks (Brashears et al. 2016), homophily preferences (McPherson et al. 2001), and preferential attachment, in the sense that alters with certain characteristics are more attractive to connect to. On the “constraints” side, we assume that individual choices are constrained by meeting opportunities (Blau 1977) or *foci* (Feld 1981), that is, social contexts that determine both the opportunity to create connections in general (e.g., someone working in a large organization may meet more people than someone in a small organization) and the opportunity to create connections with certain types of alters (e.g., a student at a university meets mostly fellow students with mostly similar social backgrounds).

Based on this general framework, we derive hypotheses on a number of potential determinants of the size (or degree) of personal WhatsApp networks. First, we expect that *degree decreases with age*, as both the cognitive abilities to create and maintain social connections may decrease as people get older, as well as their physical ability to engage with meeting opportunities (Wrzus et al. 2013). Moreover, we expect that *degree increases with level of education* (DiPrete et al. 2011; Lubbers et al. 2019), as people with more education may on average have higher cognitive abilities allowing them to maintain larger networks. Similarly, research has suggested that women are better at managing social relations (e.g., have better network recall; Brashears et al. 2016; Lubbers et al. 2019), we hypothesize that *women have larger networks than men*. Arguing from the logic of preferential attachment, *people with higher incomes may attract more connections* as their resources (of which income serves as an indicator) may be a source of social capital to others. An alternative mechanism that leads to the same prediction is that people with higher incomes are more likely to work in larger organizations, which provides them with more opportunities to form relations.

A prediction that follows from the logic of meeting opportunities is that *people with a partner and/or children have higher degrees* as they participate in more social contexts that provide meeting opportunities, such as schools, day care centers, sports clubs, etc. Lastly, the logics of preferences and meeting opportunities may also interact. Hofstra et al. (2021) argue that due to homophily preferences, people are likely to connect to others of the same ethnic background, but that due to differential group sizes, the opportunities to do so systematically differ between groups. As a result, *members of minority groups are expected to have smaller networks than members of the majority*.

Currently, there is - to our knowledge - very little research on the individual determinants of group membership in online social networks. Accordingly, we are not aware of established theoretical arguments on what drives group membership. Hence, as a starting point, we assume that the mechanisms that drive network size, as outlined above, also determine group membership. That is, we assume that while groups provide an alternative mode of communication as compared to one-to-one contacts, the *number* of groups that one participates in is driven by the same logic of choice and constraint. We thus test the same hypotheses that we specified for degree also for the number of groups. Throughout the analysis, we control for technological skills.

## Data and methods

### Data donation

In the past years, the predominant approach for accessing digital traces data has been the use of Application Programming Interfaces (APIs) of online platforms (Freelon 2018). However, these APIs are rapidly being restricted; moreover, for the specific case of Instant Messenger Services, access via APIs has hardly been available in the first place. Recently, a promising new approach to gain access to digital traces has emerged thanks to the General Data Protection Regulation (GDPR)’s right to data access by the individual on whom the data has been collected and data portability (Ausloos and Veale 2020). As a result of this legislation, all data processing entities are required to provide citizens a digital copy of their personal data that those entities collect upon citizens’ requests. These pieces of personal data provided, for example, by platforms such as Facebook, WhatsApp, Netflix, and others as machine-readable files are commonly referred to as *Data Download Packages* (DDPs). This legislation now also provides researchers with the opportunity to invite participants to share their DDPs for research purposes. Performing data access requests and then sharing DDPs for research purposes is what we refer to as *data donation*. In comparison to using trackers, data donation has the advantage of being able to collect data generated through multiple devices, can collect data in retrospect and can collect both public and private data (Breuer et al. 2022).

A major challenge when using data donation is the fact that DDPs potentially contain very sensitive data. In addition, not all data is required when answering a specific research question. To tackle this challenge, (Boeschoten et al. 2022) developed an alternative workflow consisting of the following steps:

1. The participant requests their personal DDP at the platform that is of interest to the research project.
2. The participant downloads the DDP on their own personal device after the platform has made the requested DDP available for download.
3. The participant visits a webpage tailored to the specific research project. Here, they open the DDP which enables a local processing step, meaning that only the variables of interest to the specific research project are extracted from the DDP.
4. Once the local processing step is finished, the extracted variables are shown on screen. The participant can inspect these data and decide whether they want to donate these (or decline donation).
5. Only after the participant has decided to donate, the extracted variables are sent to a server that can be accessed by the researcher.

Port is an app that facilitates the local processing step described in step 4 in a generic way, meaning that the software can be used by researchers from any institution and configured to extract variables of interest from DDPs from any platform (Boeschoten et al. 2023). Other software tools with similar functionalities, but with a less generic set-up are for example OSD2F (Araujo et al. 2022), Designerly Data Donation (Ortega et al. 2021), the Data Donation Module (Pfiffner et al. 2022) and the Social Media Donator (Zannettou et al. 2023).

As these apps are all relatively new, few academic papers utilizing them to collect donated data have appeared yet. For example, Haim et al. (2023) expanded the OSD2F tool and utilized it to collect data from Twitter, Instagram, Facebook and YouTube. Gómez Ortega et al. (2022) requested their participants to donate data from menstrual tracking apps through the Designerly Data Donation app, and Google Assistant data in another application (Ortega et al. 2023). Direct donation of complete DDPs, so without interference of any app aimed to preserve privacy prior to donation, has been used for data collection in multiple studies. For example, Van Driel et al. (2022) collected Instagram DDPs to investigate the relationship between social media use and well-being and Kmetty and Németh (2022) collected Facebook DDPs to investigate research participants’ musical preferences.

In this paper, we apply the method sketched above to WhatsApp. Specifically, we asked respondents to request their data package from WhatsApp and donate part of the data contained in the DDP for our study. WhatsApp account data can be requested from the app and contain information on the WhatsApp contacts of the user (as a list of phone numbers), the groups that the user is a member of, and some basic account information, such as the type of device, the timestamp of the latest login, and the “status message”. In the local processing stage, we extract from this package the total number of contacts and the total number of groups per respondent, after which the respondent is asked to donate these data. These are the primary outcome measures in our analysis.^1^

Before we discuss further methodological details, it is useful to highlight key limitations of user data as provided by WhatsApp. First, the data contain only information about the user who requested the data, in this case, their direct contacts and their group memberships. Importantly, the data do *not* contain information about connections *between* the contacts of the user, which (in the context of a sample from a large population) makes any analysis beyond direct connections (e.g., measuring clustering or community structure) impossible. This is analogous to the measurement of “ego-networks” (or personal networks) in large samples, which typically also lack information on indirect connections.^2^ Similarly, the DDP contains only information about the group memberships of the user, but not about other members of the group.

Second, the DDP does not contain any information on the *contents* of chat messages, for the simple reason that the service does not store these messages and is therefore not able, nor obliged by the GDPR to provide these data in a data request. While it is possible to export chat conversations on a device which could be used in a data donation study, this is (at the time of writing) only possible for one chat conversation at a time (WhatsApp 2025). This implies that collecting large numbers of chat conversations is prohibitively time-consuming (for respondents). Moreover, due to privacy concerns, collecting raw chat data is problematic, as they contain not only data about the respondent but also about conversation partners. Consequently, chat conversations will have to be processed and anonymized on the respondent’s device before donation, which, depending on the measures of interest, may require considerable computation power. This makes systematically collecting data on the contents of large numbers of conversations per respondent very challenging, even if privacy concerns can be addressed. In turn, this implies that studying the content of connections on WhatsApp, (e.g., tie strength) is currently not feasible at scale. Given these limitations, we focus on the numbers of contacts and groups, as these are currently the only measures available to study the topology of the WhatsApp network at scale.

### Sample and data collection

We collected data through the LISS panel, a Dutch probability-based online panel of the general population (Scherpenzeel and Das 2010). The LISS panel^3^ consists of approximately 7500 Dutch-speaking individuals aged 16 and older who permanently reside in private households in the Netherlands. The panel has a longitudinal component, the annual LISS Core Study, which comprises multiple topical surveys conducted each year since 2007. In addition, the panel is open for fielding contributed cross-sectional or longitudinal studies in the social and behavioral sciences. The Core Study covers a wide range of domains, including health, work, education, income, housing, leisure and time use, political views, values and personality. All data collected in the LISS panel are disseminated and made available to researchers through the LISS Data Archive. For the current study, 4,800 panel members were randomly selected and invited to participate.

Our data donation study was included as a contributed study. At the start of the monthly fieldwork period, LISS panel members received a general email informing them that new questionnaires were available. The selected sample of panel members was presented with the questionnaire for the current study, in which they answered questions about their WhatsApp use and other related digital behaviors. After these questions (and if they were not filtered out due to, for example, not using WhatsApp), respondents saw an invitation within the survey to donate their WhatsApp data. The data donation was requested in the form of two separate DDP’s. The invitation contained information about the purpose of the data donation, the procedure, a visual example of the donated data, and the additional incentive (€5 or up to €10)^4^. After agreeing to participate in this part of the study, participants were guided through the procedure described in steps 1–5 of Sect. 3.1 (details of the procedure are provided in Appendix A). Participants were then presented with a quiz assessing how well they understood the information provided about data donation. Next, they received step-by-step instructions within the survey on how to request and download the required data files from WhatsApp. Once they stored the DDP on their personal device, participants could process it locally through a tailor-made website, extracting only the number of contacts and groups the participant had on WhatsApp. These numbers were then displayed on the screen, after which participants could decide whether to donate them. We tailored the instructions to different operating systems and allowed the procedure to take place across multiple devices: the instructions were displayed on the participant’s laptop, while the actual donation occurred on their smartphone. After each step (requesting & downloading, and donating), participants were asked about their experiences with the process, specifically regarding its difficulty and issues encountered. Participants completed the process of requesting, downloading and donating their data twice: first for the DDP containing chat data, and then for the DDP containing account data. The chat DDP was available immediately, whereas the account DDP became available three days later. Three days after requesting the account DDP, participants received a reminder email prompting them to continue with the next steps of downloading and donating.

Of the 4800 LISS panel members invited to participate, 3598 responded to the survey request (AAPOR RR1 75%; AAPOR 2023). The break-off rate was 7.9% (*n* = 378). The data collection was conducted during two fieldwork periods in February and April 2023. In early February, the survey was fielded to 800 participants, with general reminders sent during the respective fieldwork period of data collection near the end of the month to those who were invited. Participants who requested a WhatsApp account package in the final three days of the fieldwork period received a three-day extension to complete their data donation.

Between the first and second fieldwork periods, several adjustments were implemented to improve the data donation process and increase participants’ willingness to donate:

- **Shortened reminder intervals.** The intervals for sending batches of email reminders after requesting the WhatsApp account data package were reduced. Instead of weekly reminder batches, reminders were sent daily, ensuring that participants received an e-mail from the LISS panel immediately after the three-day waiting period ended.
- **Improved mobile usability.** The data extraction and donation process were optimized for mobile viewing. Initially, respondents could not proceed to the extraction instructions when filling out the survey on a mobile device. In the second period, participants using smartphones were advised to fill out the survey on a desktop or laptop but were allowed to continue on their mobile devices. For these users, a direct link (URL) to the Port web application was provided in addition to the QR code.
- **Refusal conversion screens.** Refusal conversion screens were added to the survey in the second period. Participants who initially indicated they were not willing to donate their data were presented with additional information on the data donation process. The screen emphasized that no sensitive data is shared, all data processing occurs locally, participants can review their data before sharing, and an incentive is offered for each donated package. These arguments were developed based on participant feedback during the first period, specifically the most frequently cited reasons for not engaging in data donation.

In early April, the survey was fielded to the additional 4,000 participants. As before, general reminders were sent during the second half of the month. Fieldwork concluded on April 25. Participants who requested a WhatsApp account package in the final three days of the fieldwork period were again granted a three-day extension to complete their WhatsApp account data donation.

### Variables and measures

The data collected from LISS participants can be divided in two categories: *WhatsApp data* (the donated chat DDP and account DDP) and *survey data*. From the WhatsApp account information DDP, we extracted (1) the number of WhatsApp contacts as reported by WhatsApp^5^ and (2) the number of group chats the participant was a member of.

The survey included questions on the use of mobile devices, general privacy concerns, and trust in organizations that collect data. In addition, participants were asked about their use of MIMS in general and WhatsApp in particular. WhatsApp users were asked about their estimated number of contacts and group chats, as well as to provide some characteristics about their most recently used group chat (i.e., its lifetime, number of participants, and general purpose). General demographic variables (i.e., age, gender, level of education, migration background, and income level) were obtained from the LISS “Background Variables^6^” module for all invited participants.

In the analysis explaining individual variance in the number of contacts and number of groups extracted from participant’s DDPs, we used of the following variables:

- **Age**, measured in years and obtained from the LISS Background Variables.
- **Sex**, obtained from the LISS Background Variables. We restricted our analysis to a comparison between *male* and *female* participants, as the category *other* contained only four cases.
- **Net monthly income**, measured in euros and obtained from the LISS Background Variables.
- **Level of education**, measured in the categories *low*,* middle*, and *high*. Participants were classified as *low* if they had completed only primary and/or lower secondary education. The *middle* category consisted of those whose highest completed education was secondary vocational education (*MBO* in the Netherlands). Participants were classified as *high* if they had attended a university or a university of applied sciences. This variable was obtained from the LISS Background Variables.
- **Migration background**, categorized as *no migration background*, *migration background from a Western country*, or *migration background from a non-Western country*. This variable was also obtained from the LISS Background Variables.
- **Having a partner** was measured by self report and obtained from the Background Variables

- **Having children living with you** was measured by self report and obtained from Background Variables.
- **Technological skills** were measured using 6 items. The first item measured self-assessed smartphone skills on a 5-point scale from ‘beginner’ to ‘advanced’ (Keusch et al. 2019; Struminskaya et al. 2021). The remaining four items were included from a DigIQ scale by Vries et al. (2022), particularly from the section focusing on safety and control of information and devices. On a 5-point scale ranging from ‘completely untrue’ to ‘completely true’, respondents indicated whether they knew how to:
  - Protect a device against unauthorized access (e.g., using a pin code or fingerprint);
  - Protect devices against viruses;
  - Adjust privacy settings on a mobile phone or tablet;
  - Identify suspicious emails that attempt to obtain my personal data;
  - Delete the history of previously visited websites.

To verify these six items measured a single underlying construct, we conducted an exploratory factor analysis. Both the Kaiser criterion and the scree plot indicated a one-factor solution, explaining 60% of the total variance. The corresponding factor loadings are provided in Appendix B, Table 3. Additionally, we obtained a Cronbach’s alpha of 0.863 for the scale created, indicating good internal consistency. For subsequent analysis, we used the mean score of the six items as the measure of technological skills.

Table 1 provides descriptive statistics for our key variables for the participants who donated their data.

**Table 1:** Descriptive statistics of the variables used in the regression analyses (*n* = 294)

Variable	Mean (SD)/*n* (%)	Median	Range
Number of WhatsApp contacts	261.4 (223.47)	203.0	6−1783
Number of WhatsApp groups	50.22 (50.30)	35.0	1–339
Age	42.97 (*16.01*)	41.0	16–83
Sex			
~ Female	152 (51.7%)		
~ Male	142 (48.3%)		
Income	2,315 (*1*,302.08)	2,487	0–6200
Educational level			
~ Low	63 (21.4%)		
~ Middle	56 (19.0%)		
~ High	175 (59.5%)		
Migration background			
~ No migration background	251 (85.4%)		
~ Western country	24 (8.2%)		
~ Non-western country	19 (6.5%)		
Partner (Yes)	216 (73.5%)		
Living with children (Yes)	129 (43.9%)		
Technological skill	4.56 (0.53)	4.67	1–5

### Analysis

To analyze the degree distributions for the number of contacts and the number of groups, we fit the power law distribution to the data following the method proposed by Clauset et al. (2009) and as implemented in R by Gillespie (2015). Furthermore, we fit the exponential- and log-normal distributions using maximum-likelihood estimation.

To test our hypotheses on individual degree and group membership, we estimate linear regression models. Given that both dependent variables are extremely skewed (as shown below), we take the natural logarithm of each as the dependent variables. As a robustness check, we also estimated linear regression models with the raw counts as well as negative binomial regression models, but these do not lead to a better fit. For the categorical variables level of education and migration background, we include dummy variables for their respective categories in the models.

## Response and selectivity

Before we turn to our main results, we briefly discuss response and selectivity. Like other data donation studies (Pfiffner and Friemel 2023; Silber et al. 2022), our study is also potentially sensitive to selectivity at various stages of the data donation procedure. To understand to what extent our obtained sample of data donations is affected by selectivity, we first evaluate the dropout rates throughout the course of the study. Second, we evaluate how these dropout rates might be different for different demographic groups.

### Overall response rates

From 4800 members of the LISS panel who were invited to participate in the study, 3,598 (75% of the complete sample) started the study questionnaire. 408 respondents were excluded due not having a smartphone (328 respondents), not using WhatsApp (70 respondents), or dropping out (20 respondents). 3,190 respondents (66.5% of the invited sample) were eligible for data donation. Of those, 929 indicated to be willing to share their WhatsApp data through data donation (19.3% of the invited participants). Of those, 492 participants donated data from at least one DDP, making up 10.3% of the entire sample and 53% of the participants that consented to sharing their data. In total, 349 participants donated data from their WhatsApp account DDP (7.3% of participants invited, and 9.7% of participants starting the questionnaire), while 460 participants donated data from a group chat DDP (9.6% of participants invited, and 12.8% of participants starting the questionnaire). The response and sharing rates are summarized in Table 2.

**Table 2:** Response and sharing rates of whatsapp data donation

	N (percentage of sample)
Invited sample	4800 (100%)
Started questionnaire	3598 (75.0%)
Owns a smartphone	3270 (68.1%)
Uses WhatsApp	3190 (66.5%)
Willing to share WhatsApp data	929 (19.4%)
Donated WhatsApp data (any DDP)	492 (10.3%)
Donated account data	349 (7.3%)
Donated chat data	460 (9.6%)

### Selectivity in response and donation rates

Across the different stages where participants dropped out of the study, we identify two steps as crucial for understanding how selectivity in the data donation process may have affected our sample. The first step concerns the point at which WhatsApp users indicated whether they were willing to share their WhatsApp data. The second step concerns the point at which participants who had indicated their willingness to donate their WhatsApp data, actually donated the variables extracted from their WhatsApp account DDP. We explore the extent to which compliance at these two steps varies across different demographic groups.

**Fig. 1:**
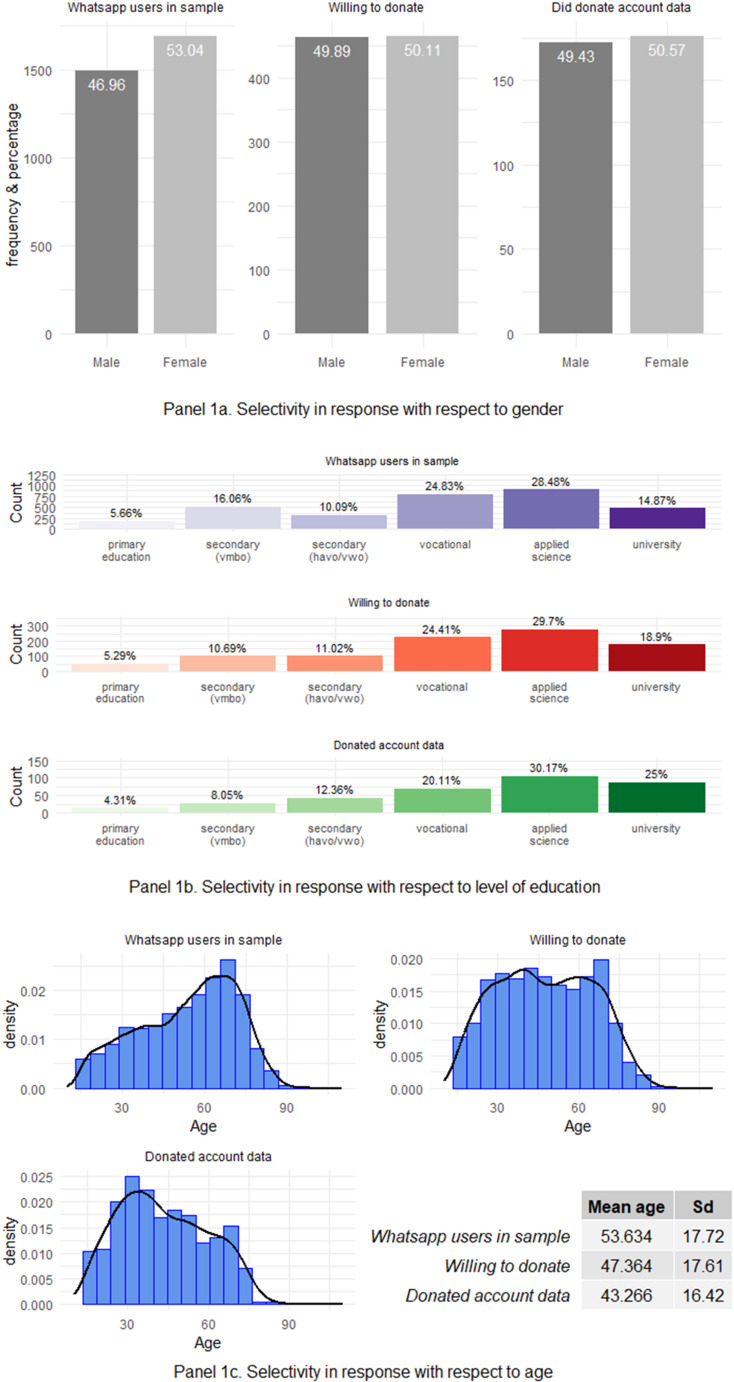
Selectivity in responses with regard to gender (**a**), level of education (**b**) and age (**c**)

Figure 1 compares the distributions of gender (Panel 1a), level of education (Panel 1b), and age (Panel 1c) among three groups: WhatsApp users in the sample, those who indicated willingness to donate, and those who actually donated their account data. For gender, we observe that while women are somewhat overrepresented (as compared to the general population) among WhatsApp users, women and men are equally willing to donate and there is no significant difference by gender among those who ultimately donated. Regarding education, we find that those with higher levels of education are somewhat more likely to both consent to donation and to actually complete the donation process. As a result, this leads to an overrepresentation of higher educated respondents among those who donated compared with the overall sample of WhatsApp users.

Finally, we find that younger users are clearly overrepresented among the respondents who donated, and that this selectivity occurs both at the consent stage and at the actual donation stage. We return to potential implications of this selectivity in the discussion section.

## Results

### Topological features

**Fig. 2:**
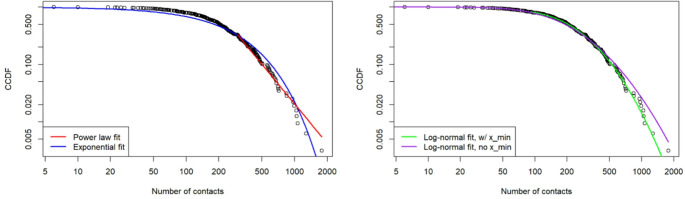
Complementary cumulative distribution of the number of WhatsApp contacts, with fitted curves for the power law- and exponential distributions (left panel) and lognormal distributions (right panel)

Figure 2 below shows the complementary cumulative distribution of the number of contacts, with the fitted curves. For the power law distribution (left panel), we estimate a lower threshold of 299 and a scaling parameter of 3.29. Clearly, this does not fit the data very well, which is confirmed by a goodness-of-fit test. Relying on the bootstrapping procedure as proposed by Clauset et al. (2009), we find that the power law fit significantly deviates from the observed distribution (*p* = 0.030). We thus reject our “naive” hypothesis that the distribution follows a power law. While the exponential distribution seems to visually fit the data much better (with an estimated rate of 0.004), a Chi-square test indicates that also this distribution significantly deviates from the observed distribution (*p* = 0.000). In contrast, the log-normal distribution (without estimating a lower threshold; µ = 5.253, σ = 0.854) does not statistically deviate from the observed distribution (Chi-square test, *p* = 0.226). When we do estimate a lower threshold (x_min_ = 91, µ = 5.402, σ = 0.698), the estimated curve visually fits the data slightly better, and indeed also this distribution does not significantly differ from the data (*p* = 0.890).

**Fig. 3:**
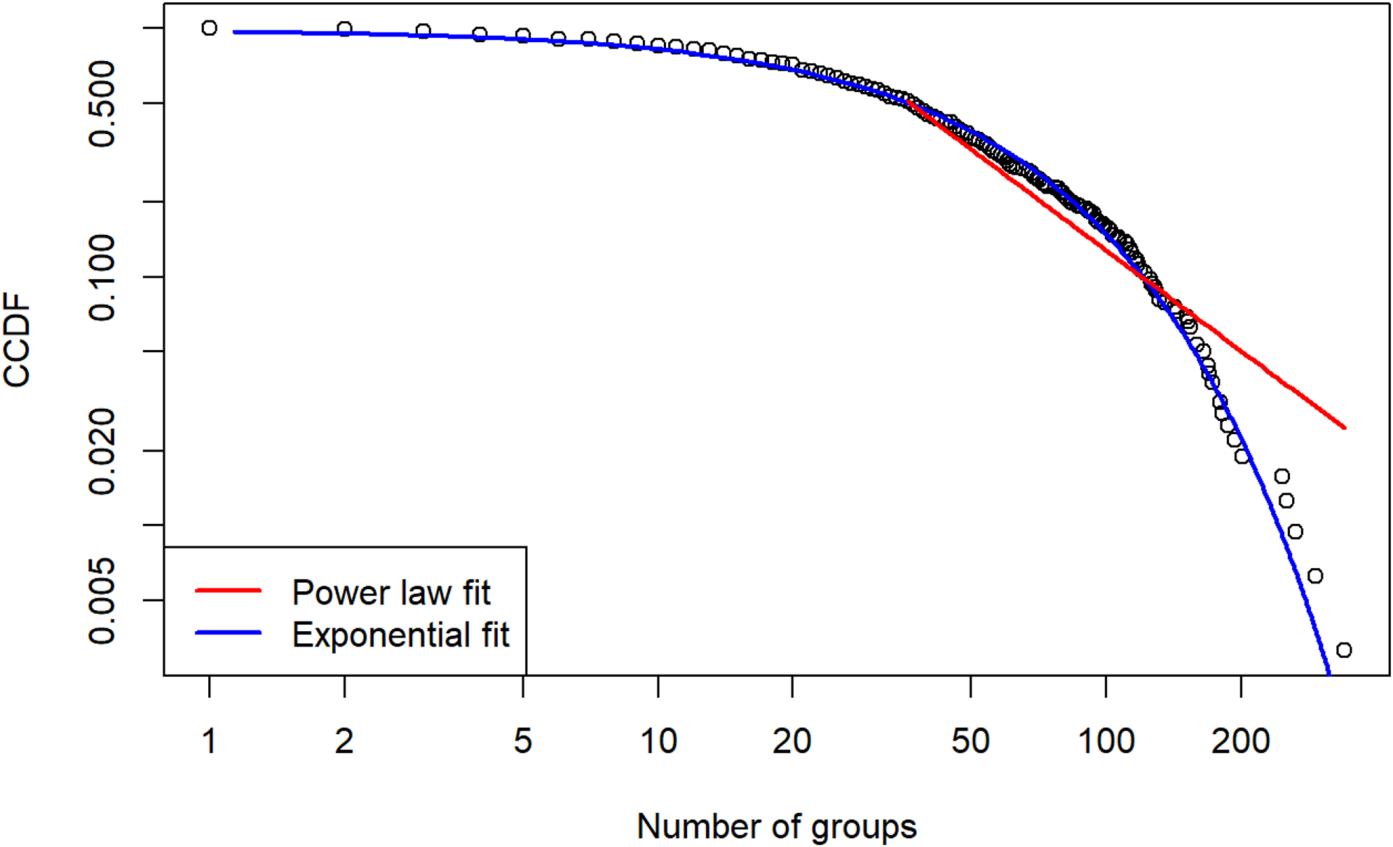
Complementary cumulative distribution of the number of WhatsApp group memberships per respondent, with fitted curves for the power law- and exponential distributions

For the distribution of groups, we also estimate the power law-, exponential-, and log-normal distribution. Figure 3 shows the results for the power law- and exponential distribution. As already becomes clear visually, the power law distribution (lower threshold = 36 and a scaling parameter of 2.34) fits the data very poorly (*p* = 0.000). The exponential distribution (*λ* = 0.019) fits the data much better and is indeed statistically indistinguishable from the observed distribution (Chi-square test, *p* = 0.802). In contrast, the lognormal distribution does deviate significantly from the data (Chi-square test, *p* = 0.006).

### Explaining individual variation

**Fig. 4:**
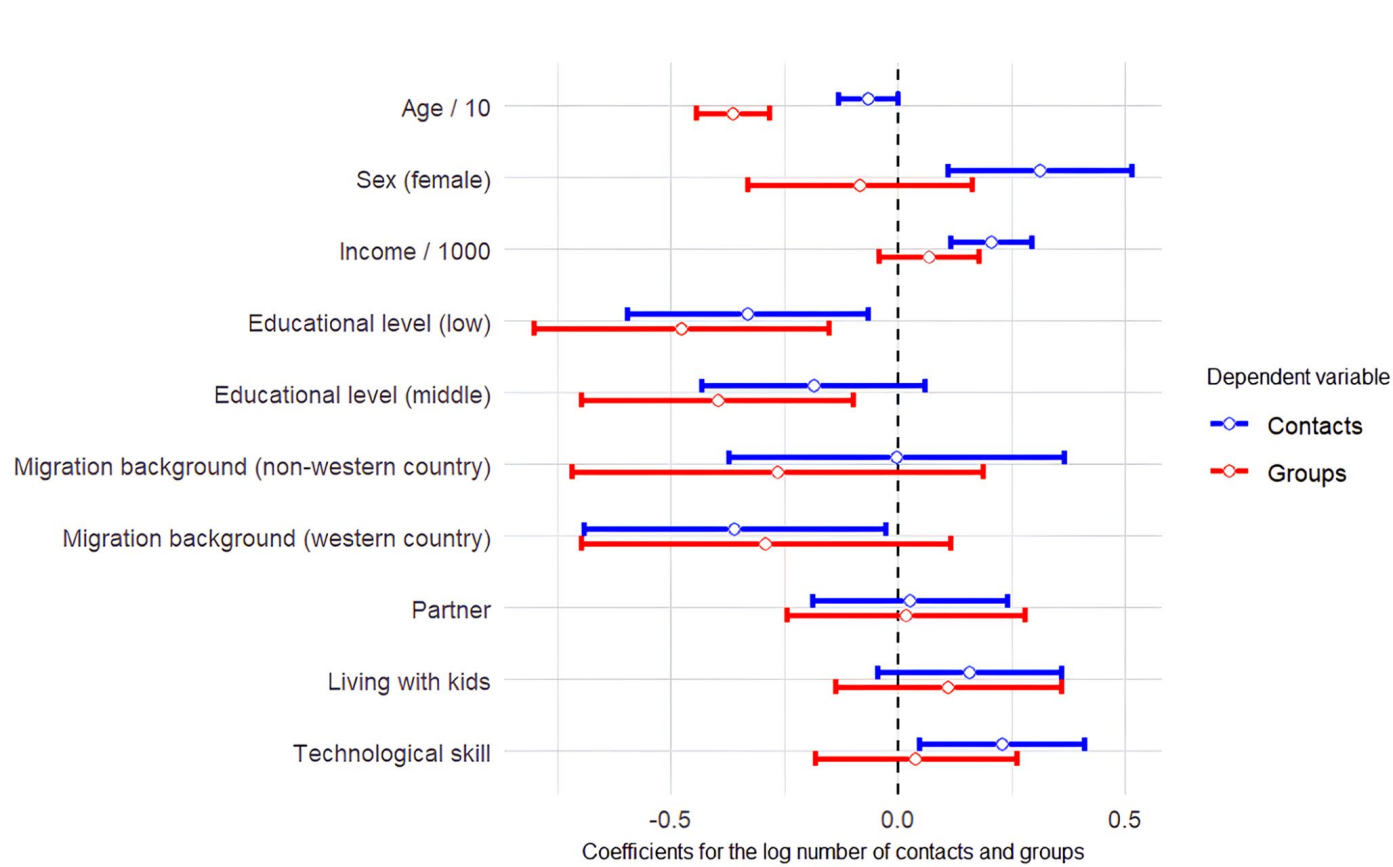
Coefficients of OLS regressions of number of contacts and number of group memberships on predictors. Bars represent 95% confidence intervals for the coefficients

Figure 4 presents the regression results for both the number of individual contacts (degree) and the number of groups (the corresponding table with the complete regression results is included in Appendix B). In line with the expectations, we find that the number of contacts significantly decreases with age, and increases with income. We furthermore find, also in line with the expectations that women have significantly more contacts, and that respondents in the lowest education category have fewer contacts than respondents in the highest education category, although the difference between the middle category and the highest category is not significant. We also find that, as expected, respondents who are more politically involved have more contacts. Contrary to our expectations however, migration background, having a partner, and having children in the household are not significantly associated with degree. Furthermore, the coefficients for these variables are consistently in the opposite direction as compared to the expectations.

Turning to the number of groups per respondent, we again find the expected associations with age and education (in this case, also the difference between the middle- and highest category is significant). However, we do not find that gender, income, migration status, relationship status, or having children are significantly associated with the number of groups.

## Conclusion and discussion

The growing popularity of Mobile Instant Messenger Services (MIMS) such as WhatsApp has not been matched by an equivalent growth with our empirical knowledge about these online social networks. Most of our knowledge about online networks is based on Twitter (now X) and Facebook. In this study, we aim to remedy this gap by applying the research strategy of *data donation* among a probability-based sample of the Dutch general population. Through data donation, research participants voluntarily provide researchers with existing digital traces that online platforms and other entities have collected about them. To our knowledge, this is the first data donation study conducted within a probability sample.

Given the lack of empirical knowledge about MIMS networks, we focused on a key feature of network topology: the distribution of the number of connections (i.e., *degree*), both at the population level and in terms of determinants of individual-level variance. Moreover, we studied the distribution of *group membership*, as the prominence of group-based communication is a defining characteristic of WhatsApp. The results can be summarized as follows.

First, we find that the distribution of connections is heavy-tailed, as expected in large networks. However, in contrast to common claims in the network science literature, we find no evidence for a power-law distribution. Instead, we find that a log-normal distribution provides the best fit to the data. Interestingly, this finding is in line with previous research claiming that large social networks, including online social networks, tend not to conform to the supposed empirical regularity of power law degree distributions in large complex networks (Corten 2012; Jackson and Rogers 2007). It is also consistent with findings from the literature on extended networks, which have likewise identified log-normal distributions (McCormick et al. 2010). Our study thereby contributes to the ongoing debate on the universality of the celebrated power law degree distribution (e.g., Broido and Clauset 2019; Voitalov et al. 2019). At the same time, however, we are not aware of an established theoretical foundation for the log-normal degree distribution in social networks, even though log-normal fits are commonly found in empirical data (Barabási 2016; Broido and Clauset 2019). Our findings therefore once more underscore the need for such theoretical foundations, that is, a plausible model of the emergence of large social networks that can account for such observed distributions.

Second, we find that the individual-to-groups degree distribution (that is, the number of group memberships per individual) also exhibits a heavy-tailed distribution. In this case, the empirical distribution appears to follow an exponential distribution. To our knowledge, this is the first study to demonstrate the heavy-tailed nature of the degree distribution in an individual-to-group network. In doing so, we contribute to the still relatively scarce empirical literature on the topology of two-mode networks (Mitrović and Tadić 2012; Shang et al. 2010; Zhang et al. 2008) and provide additional empirical evidence that can be used to theoretically model such networks (Guillaume and Latapy 2006; Vasques Filho and O’Neale 2018). More substantively, our findings reaffirm the dominance of groups in MIMS: not only do users in our data participate in, on average, more than 50 groups. Moreover, the heavy-tailed nature of the distribution also suggests that some users are extremely well-connected to groups, and may thus be able to transfer information between groups very efficiently. As compared to the influence of such “hubs” in one-mode networks (Albert and Barabási 1999), one could argue that in two-mode networks such influence is further amplified, as with each connected group users potentially reach many additional users. However, such claims warrant further investigation as such “amplification” may be dampened by overlap between the groups to which a user is connected, an aspect we could not assess with our data. Nevertheless, the large variance in degree, both for individual-to-individual and individual-to-group connections, underscores the value of examining individual-level predictors of this variance.

Turning to the explanation of individual-level variance, we find that for the number of connections, our results are to a large extent in line with the literature on extended social networks (Hofstra et al. 2021; Lubbers et al. 2019). Specifically, we find that younger users, women, those with higher incomes, and those with higher levels of education tend to have more connections, consistent with the logics of choice and constraint articulated in this literature. These findings also resonate with the general notion that online social networks are, to a large extent, reflections of interactions in face-to-face (“offline”) networks (Dunbar 2016; Dunbar et al. 2015; Hofstra et al. 2017). However, not all findings from the literature on extended networks can be reproduced: in particular, we find no significant associations with having a partner, children, or a migration background.

In our analysis of the antecedents of the number of group memberships, we tested whether the same individual traits that are expected to predict individual contacts, also predict group membership. Find that this is the case for some, but not all of these traits. Specifically, younger and higher educated respondents tend to participate in more groups. Although the associations with the remaining predictors point consistently in the same direction as for individual contacts (and consistent with expectations), none of them are statistically significant. Notably, the explained variance in the model for group membership is higher than in the model for individual connections, even though only age and education are statistically significant predictors of group membership.

Clearly, our study also has a number of limitations. First, although we started with a large panel sample, our final analytic sample was relatively small due to high drop-out rates at various stages of the data collection procedure. This illustrates an important limitation to the data donation approach: even though our procedure was specifically designed to protect participants’ privacy and despite the fact that LISS panel members are to a large extent experienced survey participants, not all respondents are willing to donate their data. However, because the methodology of data donation is still in its early stages, part of the drop-out is likely caused by technical issues or imperfections in how the procedure was communicated. Indeed, we saw that the proportion of respondents willing to donate was considerably higher than the proportion of respondents who eventually completed the donation. Nonetheless, the overall response rate in our study is on par with other data donation studies, which indicates that there is still considerable room for improvement in the methodology of data donation. As a consequence of the relatively small sample, some of the non-significant findings in our analyses may be attributed to limited statistical power.

Second, we observed considerable selectivity in response: conditional on being a WhatsApp user, younger people and more highly educated individuals were more likely to donate. While we see little reason to expect that this would bias our regression results, our descriptive findings are likely to be affected by this selectivity. That is, because our regression analyses show that younger and more highly educated individuals also tend to have more connections and group memberships, it is plausible that the shapes of our observed degree distributions are somewhat biased towards participants with higher degrees. However, this does not necessarily undermine our conclusion that these distributions are not power-law distributions, as the inclusion of more nodes with relatively low degrees (which would render the distributions more representative) would likely shift the observed distributions even further away from a power law distribution. Nevertheless, addressing such biases in order to achieve a more accurate estimation of degree distributions in the population should be a priority of future research.

Third, the meaning of the connections captured by our method is open to interpretation. The list of contacts in the DDP provided by WhatsApp includes all contacts saved in a user’s contact list who are also WhatsApp users. Consequently, we miss contacts who were not stored in the user’s contact list but with whom the user nevertheless interacted. This also implies that alters with whom the user interacted within *groups* are also not necessarily included. On the other hand, the DDP may contain contacts who are present in the user’s contact list but with whom the user never actually chatted (although they could have, since they are both WhatsApp users). These two biases work in opposite directions, and we have no reliable means of assessing their relative magnitude. A similar limitation applies to group memberships. The list of groups in the DDP contains all groups of which the user was a member at the time of the data request, regardless of whether the user (or any other group members) still actively participated in them. As a result, the list likely contains numerous “dormant” groups that remain technically active but are no longer used, simply because the user never took the initiative to delete or leave them. Clearly, the extent to which this occurs likely varies across individuals.

The seriousness of this selectivity depends largely on the nature of specific research questions posed on mobile instant messenger networks. Research that focuses on questions that relate directly to *activity* in such networks (for example, if we were to compare activity in face-to-face friendship networks to online networks) will probably require more precise assessments of which connections are actively used and which are dormant. In contrast, for research focusing on the *potential* offered by these connections (for example, the potential of mobile instant messenger networks to spread misinformation), these biases may be less consequential, as even connections that were infrequently used in the past may still be used to spread information in the future.

Nevertheless, future research should invest in improving the assessment of the nature of connections (such as tie strength), groups, and group memberships. While the data available through DDPs alone offer limited information for this purpose, the method of data donation conceptually provides the possibility to collect supplementary information, as respondents can be prompted to answer additional questions based on the data they donate. In this sense, data donation methods have an advantage over digital trace data collected via “unobtrusive” methods such as scraping or API access, as these provide little opportunity for interaction with research participants with the purpose of collecting complementary data. Furthermore, while collecting *all* chat conversations of a respondent seems unfeasible (as discussed earlier), future research could explore methods to request respondents to donate a *sample* of their chat conversations, which could then be used to assess the content and context of their connections.

Fourth, our research design only allowed the observation of direct connections of the respondent, preventing us from studying questions related to the more complex structure of the WhatsApp network, (such as clustering, community structure, or path length) that rely on indirect connections. Our study shares this limitation with much of the existing research on ego-centered networks. However, our data donation approach could, in principle, be applied to smaller, more confined populations, such as school classes or small organizations, in which all or a large fraction of the population could be asked to donate data on their connections. Conceptually, setting aside issues of nonresponse and missing data, such data could be used to reconstruct complete networks within these smaller populations and answer more *structural* questions such as the ones sketched above. We note, however, that such questions are fundamentally different from ours, as our aim was to assess properties of the overall WhatsApp network in the general population rather than within a relatively small subpopulation such as a single organization.

Fifth, our results are limited to the Netherlands. We have no ex-ante reasons to expect that the WhatsApp networks in other national populations would be fundamentally different, and as such, we believe our results provide a valuable first insight into the nature of instant messenger networks. Nevertheless, a more comparative approach to empirically assess any national differences, or even the structure of the *global* WhatsApp network would be an interesting direction for future research. Our approach and the underlying technology would, in principle, be scalable to multiple countries, although this would require considerably more resources (and investments in infrastructure) than were available for the present study.

Another promising avenue for further research concerns studying the actual content of chat messages via data donation. Although the collection of chat messages is conceptually possible, it faces considerable practical and ethical challenges. First, due to the nature of the platform, the WhatsApp DDP does not contain chat messages (as these data are not stored by the platform in the first place). While the smartphone app allows for exporting individual chat conversations, this can only be done one at a time, making the large scale collection of chat conversations prohibitively time-consuming for respondents. Second, due to privacy concerns, collecting raw chat data is problematic, as they contain not only data about the respondent but also about conversation partners. Consequently, chat conversations would need to be processed and anonymized on the respondent’s device prior to donation, which, depending on the measures of interest, may require considerable computational resources. Nevertheless, our study demonstrates that data donation provides a valuable and feasible method for the empirical study of online platforms that have thus far remained largely inaccessible to researchers.

## Supplementary Information

Below is the link to the electronic supplementary material.

Supplementary Material 1

## Data Availability

The data collected for this study are available in the LISS data archive, 10.57990/ge66-3v89. Code for replication is available at https://osf.io/tcjb3/.

## Appendix A: the data donation procedure

This appendix provides a detailed account of the study flow, outlining the step-by-step process as experienced from the participant’s perspective.

LISS panel members sampled for the study received an email invitation from the LISS panel to complete a survey, as they do every first Monday of each month. Participants first logged in to their personal online panel environment and completed the questionnaire following the standard procedure familiar to them. The questionnaire consisted of several topical modules, including questions on mobile device use, digital literacy, privacy concerns, WhatsApp use, as well as an invitation to participate in the data donation part of the study. Respondents were screened out at different stages of the survey: those who did not use a smartphone did not proceed to the device use questions; those who did not use WhatsApp were screened out before the WhatsApp related questions were asked. Only respondents who owned an iPhone or an Android smartphone and used WhatsApp continued to the data donation section of the study.

Embedded within the questionnaire, the data donation request consisted of several parts: (1) the information about the research questions that could be addressed with data that WhatsApp collects about participants, (2) a description of the data donation procedure outlining the steps participants would need to take to donate their data, and (3) an example of the extracted data, along with information about an additional incentive participants would receive if they agreed to and successfully donated their data (€5 for one DDP or €10 for two DDPs). After reviewing this information, survey respondents were asked whether they were willing to participate in the data donation part of the study. In the subsequent question, participants were presented with knowledge questions assessing how well they understood the information provided about data donation. At this stage, participants completing the questionnaire on a smartphone were advised to switch to a PC or laptop, as they needed to follow instructions on that device while being guided through the process of requesting and saving their DDPon their smartphone.

Participants who indicated that they were not willing to participate in the data donation study were screened out at this point, while participants who agreed to participate were presented with detailed, step-by-step instructions on how to export a WhatsApp group chat history and store it on their smartphone.^7^ The instructions were tailored to the participant’s smartphone operating system (Android or iOS). After completing these steps, participants were asked about their experience with the process, specifically regarding its level of difficulty and any issues they encountered.

Participants who successfully completed the steps of requesting their data from WhatsApp and saving it on their smartphone were then presented with a QR code on their screen. Scanning this QR code with their smartphone, opened the data donation web application Port in their mobile browser. Within Port, participants first viewed an information screen, followed by a screen containing a button to select their exported WhatsApp group chat file from their device. At this stage, a local processing step took place, during which only the datapoints of interest were extracted from the WhatsApp chat export (as explained in Sect. 3.1). Specifically, the names of all group chat members were temporarily extracted and displayed to the participant, who was asked to identify which name corresponded to their own. This information was used solely to link the correct data to the participant. Immediately afterward, Port deleted the list of names; none of this information was stored or sent. Once the local processing was complete, the extracted data were displayed on screen. Participants could review the data then choose whether to donate it by selecting either the *‘Yes*,* donate’* or *‘No’* button. Only if the participant selected *‘Yes*,* donate’*, the extracted data were transmitted to the same secure server where their questionnaire responses are stored.

Once participants completed the first data donation procedure, they returned to the questionnaire environment on their laptop or computer. There, they received instructions to also request their WhatsApp account data on their smartphone. After submitting this request, it took WhatsApp up to three days to prepare the data export. During this period, participants paused their participation, and the LISS panel sent them a reminder email after three days. When participants opened the reminder, they resumed the questionnaire from where they had left off. At that point, they again saw a QR code, which they could scan to perform the data donation step, following the same procedure described earlier for the WhatsApp group chat export.

The Port application was hosted by Eyra^8^ on an AWS server. No data were stored on Port at any point, and a data processing agreement was in place between Eyra and Centerdata (which manages the LISS panel).

## Appendix B: additional results

See Tables 3 and 4.

**Table 3:** Factor loadings in the exploratory factor analysis for the constructed scale on technological skill

Item	Factor loading
Self assessment of smartphone skills	0.643
I know how to protect a device against access (e.g., a pin code or a fingerprint)	0.707
I know how to protect devices against viruses	0.822
I know how to adjust the privacy settings on a mobile phone or tablet	0.878
I know how to identify suspicious emails that try to get my personal data	0.777
I know how to delete the history of websites that I have visited before	0.791

**Table 4:** Regression outcomes when predicting the number of contacts and groups extracted

	Log extracted contacts	Log extracted groups
Intercept	3.90*** (0.504)	4.84*** (0.617)
Age/10	− 0.066* (0.033)	− 0.363*** (0.041)
Female^a^	0.312** (0.102)	− 0.083 (0.125)
Income / 1000	0.204*** (0.046)	0.068 (0.056)
Education_low^b^	− 0.330* (0.135)	− 0.475** (0.165)
Education_middle^b^	− 0.185 (0.124)	− 0.397** (0.152)
Migration background – non-western countries^c^	0.003 (0.188)	− 0.266 (0.230)
Migration background – western countries^c^	− 0.359* (0.169)	− 0.291 (0.207)
^d^ Partner (yes)	0.026 (0.109)	0.017 (0.134)
^e^ Children living with you (yes)	0.157 (0.103)	0.111 (0.126)
Technological skill	0.229* (0.092)	0.039 (0.113)
Number of observations	294	294
Adjusted R^2^	0.171	0.262
F-statistic (df = 10, 283)	7.037***	11.380***

^a^Reference category = male

^b^Reference category = high education

^c^Reference category = no migration background

^d^Reference category = has a partner

^e^Reference category = has children living with them

****p* < 0.001, ***p < 0.01*, **p < 0.05*

## Footnotes

1 As part of the data collection process, we also collected a limited amount of data from chat conversations. We do not analyse these data in this paper.

2 Ego-network data sometimes include measures of relations between “alters”, but these are then based on the respondent’s *perception* of these relations.

3
https://lissdata.nl.

4 Half of the respondents were randomly selected to view a visual example of the donated data as part of a separate methodological study on data donation.We do not discuss the results of these experiments in this paper.

5 Listed as WhatsApp contacts are phone numbers saved in the respondent’s phone’s contact list that are also reachable via WhatsApp (i.e., the owner is a WhatsApp user). Not included are (1) contacts who are not WhatsApp users and (2) people with whom the respondent has exchanged messages but who are not saved in their phone’s contact list.

6 10.57990/qn3k-as78.
