# Supplementary material for: Assessing mobile instant messenger networks with donated data
Source: Soc Netw Anal Min. 2025 Dec 27;16(1):23. doi: 10.1007/s13278-025-01550-8 (PMC12819457; doi:10.1007/s13278-025-01550-8)
Supplement: Supplementary file 1 — Supplementary Material 1 [file 13278_2025_1550_MOESM1_ESM.docx]

# Appendix A: The data donation procedure

This appendix provides a detailed account of the study flow, outlining the step-by-step process as experienced from the participant’s perspective.

LISS panel members sampled for the study received an email invitation from the LISS panel to complete a survey, as they do every first Monday of each month. Participants first logged in to their personal online panel environment and completed the questionnaire following the standard procedure familiar to them. The questionnaire consisted of several topical modules, including questions on mobile device use, digital literacy, privacy concerns, WhatsApp use, as well as an invitation to participate in the data donation part of the study. Respondents were screened out at different stages of the survey: those who did not use a smartphone did not proceed to the device use questions; those who did not use WhatsApp were screened out before the WhatsApp related questions were asked. Only respondents who owned an iPhone or an Android smartphone and used WhatsApp continued to the data donation section of the study.

Embedded within the questionnaire, the data donation request consisted of several parts: (1) the information about the research questions that could be addressed with data that WhatsApp collects about participants, (2) a description of the data donation procedure outlining the steps participants would need to take to donate their data, and (3) an example of the extracted data, along with information about an additional incentive participants would receive if they agreed to and successfully donated their data (€5 for one DDP or €10 for two DDPs). After reviewing this information, survey respondents were asked whether they were willing to participate in the data donation part of the study. In the subsequent question, participants were presented with knowledge questions assessing how well they understood the information provided about data donation. At this stage, participants completing the questionnaire on a smartphone were advised to switch to a PC or laptop , as they needed to follow instructions on that device while being guided through the process of requesting and saving their DDPon their smartphone.

Participants who indicated that they were not willing to participate in the data donation study were screened out at this point, while participants who agreed to participate were presented with detailed, step-by-step instructions on how to export a WhatsApp group chat history and store it on their smartphone.^^[[1]](#footnote-1)^^ The instructions were tailored to the participant’s smartphone operating system (Android or iOS). After completing these steps, participants were asked about their experience with the process, specifically regarding its level of difficulty and any issues they encountered.

Participants who successfully completed the steps of requesting their data from WhatsApp and saving it on their smartphone were then presented with a QR code on their screen. Scanning this QR code with their smartphone, opened the data donation web application Port in their mobile browser. Within Port, participants first viewed an information screen, followed by a screen containing a button to select their exported WhatsApp group chat file from their device. At this stage, a local processing step took place, during which only the datapoints of interest were extracted from the WhatsApp chat export (as explained in Section 3.1). Specifically, the names of all group chat members were temporarily extracted and displayed to the participant, who was asked to identify which name corresponded to their own.. This information was used solely to link the correct data to the participant. Immediately afterward, Port deleted the list of names; none of this information was stored or sent. Once the local processing was complete, the extracted data were displayed on screen. Participants could review the data then choose whether to donate it by selecting either the *‘Yes, donate’* or *‘No’* button. Only if the participant selected *‘Yes, donate’* , the extracted data were transmitted to the same secure server where their questionnaire responses are stored.

Once participants completed the first data donation procedure, they returned to the questionnaire environment on their laptop or computer. There, they received instructions to also request their WhatsApp account data on their smartphone. After submitting this request, it took WhatsApp up to three days to prepare the data export. During this period, participants paused their participation, and the LISS panel sent them a reminder email after three days. When participants opened the reminder, they resumed the questionnaire from where they had left off. At that point, they again saw a QR code, which they could scan to perform the data donation step, following the same procedure described earlier for the WhatsApp group chat export.

The Port application was hosted by Eyra^^[[2]](#footnote-2)^^ on an AWS server. No data were stored on Port at any point, and a data processing agreement was in place between Eyra and Centerdata (which manages the LISS panel).

#

# Appendix B: Additional results

Table B1.
Factor loadings in the exploratory factor analysis for the constructed scale on technological skill.

| **Item** | **Factor loading** |
| --- | --- |
| Self assessment of smartphone skills | .643 |
| I know how to protect a device against access (e.g., a pin code or a fingerprint) | .707 |
| I know how to protect devices against viruses | .822 |
| I know how to adjust the privacy settings on a mobile phone or tablet | .878 |
| I know how to identify suspicious emails that try to get my personal data | .777 |
| I know how to delete the history of websites that I have visited before | .791 |

Table B2.
Regression outcomes when predicting the number of contacts and groups extracted.

|  | *Log extracted  contacts* | *Log extracted groups* |
| --- | --- | --- |
| *Intercept* | 3.90**^***^** (.504) | 4.84**^***^** (.617) |
| *Age / 10* | -.066**^*^** (.033) | -.363**^***^** (.041) |
| *Female^a^* | .312**^**^** (.102) | -.083 (.125) |
| *Income / 1,000* | .204**^***^** (.046) | .068 (.056) |
| *Education_low^b^* | -.330**^*^** (.135) | -.475**^**^** (.165) |
| *Education_middle^b^* | -.185 (.124) | -.397**^**^** (.152) |
| *Migration background – non-western countries^c^* | .003 (.188) | -.266 (.230) |
| *Migration background – western countries^c^* | -.359^*^ (.169) | -.291 (.207) |
| *Partner (yes)* | .026 (.109) | .017 (.134) |
| *Children living with you (yes)* | .157 (.103) | .111 (.126) |
| *Technological skill* | .229**^*^** (.092) | .039 (.113) |
| Number of observations | 294 | 294 |
| Adjusted R^2^ | .171 | .262 |
| F-statistic (df = 10, 283) | 7.037^***^ | 11.380^***^ |

^a^reference category = male; ^b^reference category = high education;
^c^reference category = no migration background; ^d^reference category = has a partner;
^e^reference category = has children living with them
 **^***^** *p* < .001, **^**^** *p < .01, ^*^ p < .05*

1. The chat history data are not analyzed in this paper. [↑](#footnote-ref-1)
2. https://www.eyra.co/ [↑](#footnote-ref-2)
